# Comparison of Artificial Intelligence Detection and Short-Term Breast Cancer Risk Models within and beyond Their Intended Use in Mammography Screening

**DOI:** 10.1148/rycan.250609

**Published:** 2026-09-25

**Authors:** Yao-Kuan Wang, Lesley Cockmartin, Tobias Wagner, Zan Klanecek, Nicholas Marshall, Valerie Celis, Chantal Van Ongeval, Andrej Studen, Robert Jeraj, Hilde Bosmans

**Affiliations:** ^1^Department of Imaging and Pathology, KU Leuven, Leuven, Belgium; ^2^Department of Radiology, University Hospital Leuven, Herestraat 49, Box 7003, 3000 Leuven, Belgium; ^3^Faculty of Mathematics and Physics, University of Ljubljana, Ljubljana, Slovenia; ^4^Jožef Stefan Institute, Ljubljana, Slovenia; ^5^Department of Medical Physics, University of Wisconsin–Madison, Madison, Wis

**Keywords:** Mammography, Breast, Computer Applications, Detection/Diagnosis, Screening, Technology Assessment, Model Validation

## Abstract

**Purpose:**

To compare artificial intelligence–based breast cancer detection and short-term breast cancer risk prediction models in their intended and nonintended settings over a single mammography screening round.

**Materials and Methods:**

In this retrospective study, the Cohort of Screen-Age Women–Case Control dataset from Sweden (May 2008–December 2016; Hologic) was used, including patients with screen-detected and interval cancers, with interval cancers defined as a diagnosis more than 60 days after screening. Two examination-based settings were evaluated: detection (screening examinations) and short-term risk (screen-negative examinations). Mirai Risk, RSNA Detection (2023 challenge winner), Transpara Detection, and Transpara Risk models were assessed in both intended and nonintended settings. Discriminative performance was evaluated using the area under the receiver operating characteristic curve (AUC), and then clinically relevant sensitivity and specificity thresholds were compared.

**Results:**

A total of 20 187 examinations (7430 women) were included, with 741 examinations leading to diagnosis within 2 years (524 screen-detected and 217 interval cancers) and 19 446 examinations without cancer. Transpara Detection and Transpara Risk achieved similar performance for detection (AUC for each, 0.92; 95% CI: 0.91, 0.93; *P* = .93) and outperformed the other models (all *P* < .001). Transpara Detection demonstrated the highest specificity (97.1%; 95% CI: 96.8, 97.3) at double-reading sensitivity (all *P* < .001). There was no evidence of a difference in specificity of Transpara Risk and RSNA Detection at this sensitivity (*P* = .29). For risk, Transpara Risk performed best (AUC, 0.81; 95% CI: 0.78, 0.84), with 49.8% sensitivity at 90% specificity (all *P* ≤ .002) for interval cancers. There was no evidence of a difference in AUC between Mirai Risk and Transpara Detection (*P* = .96).

**Conclusion:**

Artificial intelligence–based mammographic detection and short-term breast cancer risk prediction models performed best in their intended settings.

**Keywords:** Mammography, Breast, Computer Applications, Detection/Diagnosis, Screening, Technology Assessment, Model Validation

*Supplemental material is available for this article.*

© The Author(s) 2026. Published by the Radiological Society of North America under a CC BY 4.0 license.

## Graphical abstract

**Figure:**
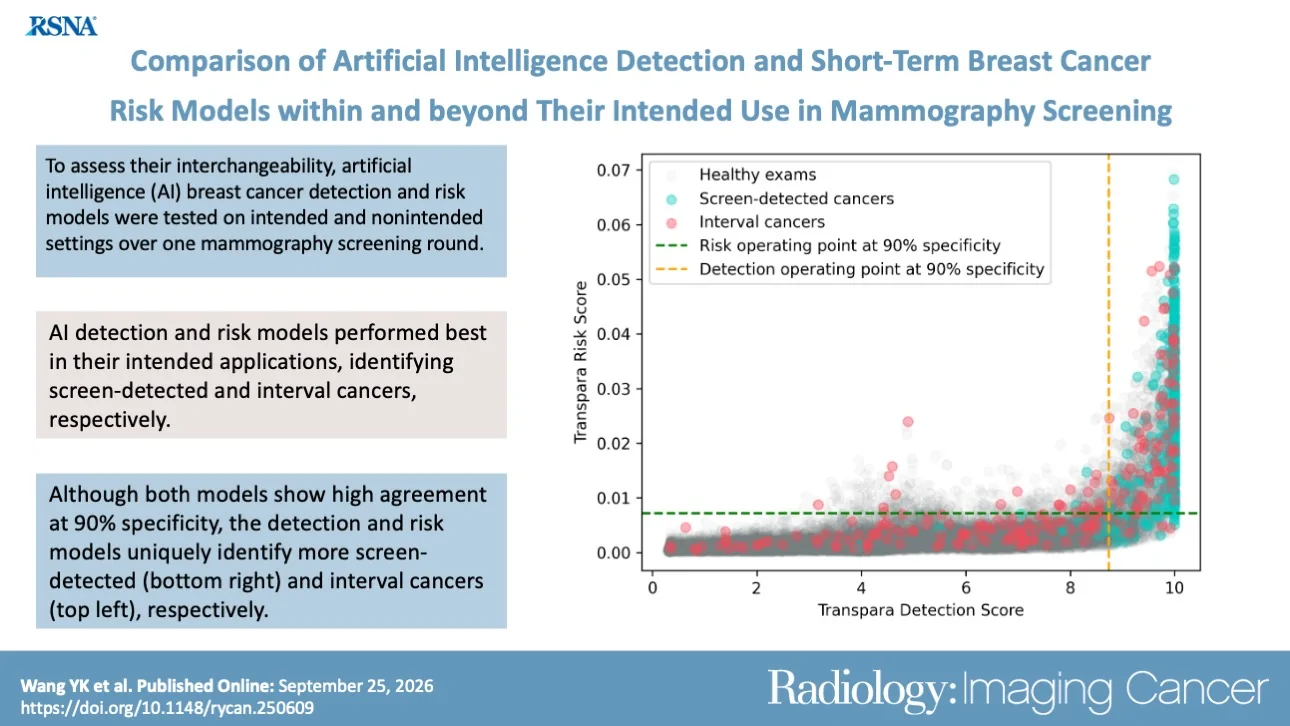

**Summary**

Artificial intelligence–based mammographic detection and short-term breast cancer risk prediction models performed best in their intended applications, with lower performance when applied outside these settings.

**Key Points**

■ In this retrospective case-control study of 20 187 screening examinations (7430 women), artificial intelligence–based mammographic detection and breast cancer risk prediction models were compared in both intended and nonintended applications using single-round screening outcomes.
■ In the screening population, detection and risk models both achieved similar performance for breast cancer detection (area under the receiver operating characteristic curve, 0.92 for both; *P* = .93), with the detection model demonstrating higher specificity (97.1%) at double-reading sensitivity.
■ In the screen-negative population, the risk model achieved the highest performance for interval cancer prediction (area under the receiver operating characteristic curve, 0.81), with 49.8% sensitivity at 90% specificity (all *P* ≤ .002).

## Introduction

Although breast cancer screening using mammography has successfully reduced mortality (1), up to one-third of breast cancers in Europe are still missed during regular screening (2), falling into the category of interval cancers. Recent research has focused on stratifying women at high risk for supplemental screening after negative results (3), aiming to detect lesions that are either difficult to identify at mammography or grow faster than the screening interval (4). Epidemiologic risk models have been developed to predict 5-year to lifetime risk on the basis of genetic mutations, reproductive risk factors, and mammographic breast density (5,6). However, their suboptimal performance and inability to predict short-term risk limit their applicability in clinical practice (7).

Artificial intelligence (AI)–based mammographic risk models have been developed to predict short-term risk (up to 5 years) from mammographic features beyond breast density (8,9) and have demonstrated better performance than traditional epidemiologic models (10,11). Promising AI models are used to either identify mammographic risk factors from large-scale screening data in a black-box manner (10) or separately account for different mammographic risk factors, such as lesion malignancy tendency and texture features (12,13). Despite the superior performance of AI models, the risk factors learned by these models are often unknown to varying degrees, making it difficult to interpret their predictions.

The aim of cancer detection is to identify suspicious lesions, whereas the aim of risk prediction is to assess future cancer risk in healthy women. However, recent studies have demonstrated the overlapping nature of these two tasks (14), which arises from missed or subtle signs in prior negative examinations (4,15) and the elevated risk associated with benign breast diseases (16,17). In addition, AI studies have reported that including lesion signs during risk model training can bias the learned features toward these easily recognizable markers, potentially causing the model to overlook subtler and more complex texture patterns (18,19). Thus, it remains unclear whether short-term risk models offer added value over detection models for risk prediction or whether risk models can replace detection models for lesion detection.

Focusing on outcomes from a single mammography screening round, this study aimed to compare AI-based breast cancer detection and short-term risk prediction models in their intended and nonintended settings to assess task-specific performance. Model performance was evaluated for detection (all screening examinations) and short-term risk prediction (screen-negative examinations only).

## Materials and Methods

### Study Cohort and Design

This retrospective study was exempt from institutional review board review due to the use of a publicly available dataset: the Cohort of Screen-Age Women–Case Control dataset (20,21), which includes screening mammograms from women age 40–74 years in Sweden (May 2008–December 2016). The dataset includes data from women with first-time diagnoses of in situ or invasive breast cancer and healthy controls whose most recent screening outcomes were negative. Prior screen-negative examinations of women from both groups are provided. All screening examinations included four standard views in full-field digital mammography, acquired using Hologic devices. The time to diagnosis is grouped as less than 60 days, 60–729 days, or more than 730 days (up to 7 years) by the dataset provider. Given that the screening intervals ranged from 18 to 24 months, screen-detected cancers were defined as examinations for which the diagnosis was made in less than 60 days and interval cancers as examinations for which the diagnosis was made in 60–729 days. All other examinations in the patient group were considered cancer priors. Examinations from patients with cancer in the 60- to 729-day category that had a subsequent screening examination were not considered as having interval cancers.

Following the original dataset curation, an examination-based case-control study design with outcomes of a single screening round was adopted for the primary analysis. Examinations with inconsistent metadata, those that could not be processed by the AI models, or those for which 2-year follow-up data were missing were excluded. Two clinically distinct settings were investigated: detection and short-term risk. In the detection setting, all examinations interpreted by radiologists in screening practice were considered, with screen-detected and interval cancers forming the case group and healthy examinations forming the control group. In the short-term risk setting, only interval cancers were included in the case group, and healthy examinations were included in the control group, representing the screen-negative population for risk prediction. Cancer priors were regarded as healthy controls in both settings. Owing to the overlap between the settings in terms of missed cancers and subtle lesions, AI models for lesion detection and risk prediction were evaluated in both settings. To better understand the performance in the risk setting, the secondary analysis focused on a long-term risk setting, in which the case group included only examinations identified as cancer priors, and the control group included examinations from healthy individuals with at least 7 years of cancer-free follow-up.

### Lesion Detection and Risk Prediction Models

Four mammogram-based AI models designed for lesion detection or risk prediction were used: the publicly available Mirai Risk (10,11,22), the winning model (RSNA Detection) (23) from the 2023 RSNA Screening Mammography Breast Cancer Detection AI Challenge (24), and the commercially available Transpara Detection and Transpara Risk models (version 2.1.0 and research version 1.0.1; ScreenPoint Medical) (13,25). None of these models were trained with the Cohort of Screen-Age Women–Case Control dataset. No financial support was provided by ScreenPoint Medical. Mirai Risk and RSNA Detection were trained on data from screening populations with screen-detected and interval cancers, although Mirai Risk is not designed for lesion detection. Mirai Risk predicts cancer presence over 1–5 years, whereas RSNA Detection predicts cancer diagnosis within a 2-year timeframe. In this study, both publicly available models used only mammograms as inputs.

Unlike the publicly available models, which were trained in a fully black-box manner, the Transpara models first extract clinically meaningful mammographic features and then aggregate them for prediction. Transpara Detection, which is optimized for screening examinations, identifies suspicious regions within mammograms and assigns detection scores on the basis of these detected lesions. Transpara Risk (improved from the previous version [13,25]), which is trained only on screen-negative examinations, first calculates breast texture scores, which are correlated with inherent risk and are independent of lesion bias. The texture score is combined with the Transpara Detection score and Transpara breast density to produce 2- to 5-year risk scores. For the Transpara models, additional data imputation for age and compressed breast thickness was necessary because the Cohort of Screen-Age Women–Case Control dataset was anonymized (detailed in Appendix S1).

Although Mirai Risk and Transpara Risk provide risk scores for up to 5 years, only 2-year risk prediction was considered in this study to focus on the overlapping timeframe for lesion detection and risk prediction. Because breast density is a promising clinical metric for selecting women who may require supplemental screening, the Transpara volumetric breast density model was included as a reference in both risk settings.

### Selection of Operating Points

Operating points were selected on the basis of clinically relevant metrics specific to two settings in the primary analysis to compare sensitivity and specificity. For the detection setting, all the models were matched to the double-reading sensitivity of the radiologists, as calculated from this dataset to enable comparison with human performance. Models were further characterized by their ratio of flagged screen-detected to interval cancers at this sensitivity level, illustrating each model’s tendency to flag different cancer types. Operating points were also considered at 90% specificity to align with U.S. screening practices, although the accompanying recall rate is not acceptable in the European context (26). For the short-term risk setting, operating points were set to achieve 90% specificity, resulting in approximately 10% of women being classified as high risk and recommended for supplemental MRI screening (27). Finally, the agreement between the best detection and risk models for screen-detected and interval cancers was evaluated, using a 90% specificity operating point for both models.

### Statistical Analysis

Performance was evaluated primarily using the area under the receiver operating characteristic curve (AUC). Subgroup analyses were performed across invasive and in situ cancers, and AUCs were reported. The DeLong method was used to estimate CIs for all AUCs and differences for paired AUCs. Bootstrap (1000 iterations) was used for unpaired AUC differences in the subgroup analyses. Differences in sensitivity and specificity were assessed using the McNemar test, and their CIs were calculated using the Wilson score method. The CIs for the ratio of screen-detected to interval cancers (at the double-reading sensitivity) were calculated using bootstrapping, as described above. All tests were two-sided, with a *P* value less than .05 indicating statistical significance. For each primary metric (AUC, sensitivity, and specificity), *P* values were adjusted for multiple comparisons using the Benjamini-Hochberg procedure across six pairwise tests within the four AI models. All the metrics were reported with 95% CIs. Statistical analyses were performed using Python (version 3.12.12; Python Software Foundation) and the Python packages NumPy 2.4.1, SciPy 1.17.0, and Statsmodels 0.14.6. All code is available at *https://gitlab.kuleuven.be/medphysqa/deploy/ai-cad-vs-breast-cancer-risk-models*.

## Results

### Demographics and Screening Characteristics

A total of 24 697 examinations from 8723 women are included in the Cohort of Screen-Age Women–Case Control dataset. After exclusions, 20 187 examinations from 7430 women were included in the analysis (Fig 1). The available ages at examination were anonymized into two groups: 11 401 and 8786 examinations were performed in women age 40–55 years and women older than 55 years, respectively. For the primary analysis with a single-round outcome, 741 examinations (741 women) led to a cancer diagnosis within 2 years (78 in situ, 650 invasive, and 13 unknown), whereas 19 446 examinations (7147 women) remained healthy. Among the cancer examinations, 524 and 217 examinations resulted in screen-detected and interval cancers, respectively, yielding a double-reading sensitivity of 70.7% (524 of 741 [95% CI: 67.3, 73.9]). For the secondary long-term risk analysis, 1085 examinations from 590 women resulted in cancer priors up to 7 years, and healthy outcomes were obtained for 4058 examinations from 4051 women. Other demographic details are summarized in Tables 1 and 2.

**Figure 1:**
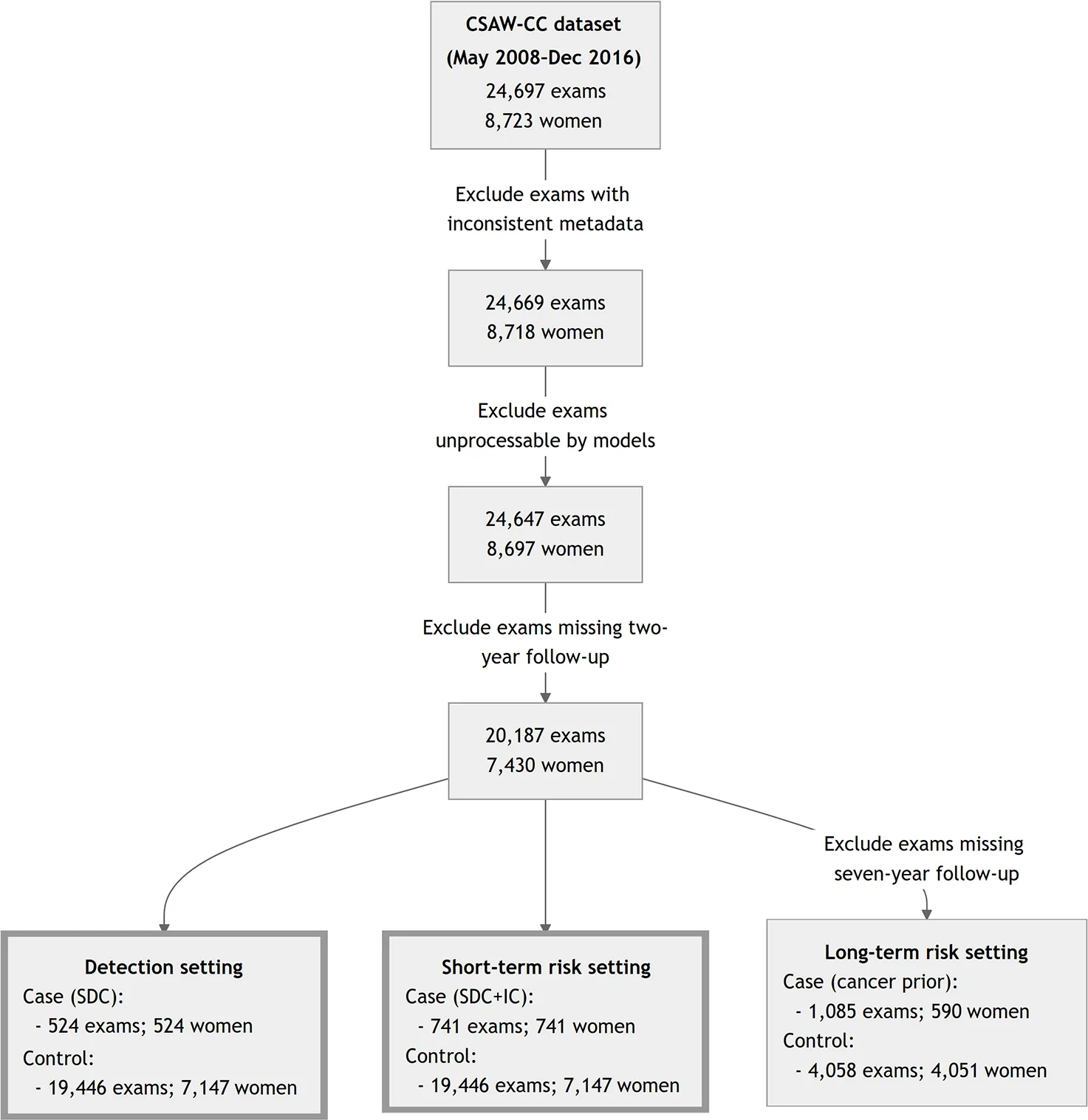
Flow chart shows the data exclusion for the publicly available Cohort of Screen-Age Women–Case Control (CSAW-CC) dataset. Examinations with inconsistent metadata, those that could not be processed by the artificial intelligence models, or those for whom 2-year follow-up data were missing were excluded from this study. The primary analysis (thick borders) involved two settings with outcomes from a single screening round: detection and short-term risk. In the detection setting, examinations resulting in screen-detected cancers (SDCs) or interval cancers (ICs) were considered positive cases; in the short-term risk setting, positive cases were defined exclusively as ICs. As a reference, a secondary analysis of the long-term risk setting was included, in which positive cases were defined as examinations followed by a breast cancer diagnosis occurring after at least one screening round and up to 7 years after the initial examination.

**Table 1:** Examination-based Demographics for Detection and Short-Term Risk Settings—Primary Analysis

Demographic	Screen-Detected Cancer	Interval Cancer	Healthy
Age (y)			
40–55	158/524 (30.2)	102/217 (47.0)	11 141/19 446 (57.3)
>55	366/524 (69.8)	115/217 (53.0)	8305/19 446 (42.7)
Density			
BI-RADS A	9/524 (1.7)	3/217 (1.4)	445/19 446 (2.3)
BI-RADS B	245/524 (46.8)	37/217 (17.1)	7668/19 446 (39.4)
BI-RADS C	237/524 (45.2)	139/217 (64.1)	9741/19 446 (50.1)
BI-RADS D	33/524 (6.3)	38/217 (17.5)	1592/19 446 (8.2)
Biopsy			
Invasive	459/524 (87.6)	191/217 (88.0)	0
In situ	62/524 (11.8)	16/217 (7.4)	0
Unknown	3/524 (0.6)	10/217 (4.6)	0
Total			
Examinations	524	217	19 446
Women	524	217	7147

Note.—Data are proportion of examinations with percentage in parentheses. Breast Imaging Reporting and Data System (BI-RADS) density was assessed using Transpara software. Examinations of screen-detected and interval cancers directly led to diagnosis within 2 years. Owing to the examination-based evaluation design, other prior negative examinations from women who later developed cancer were considered healthy controls, resulting in some women overlapping between the case and control groups.

**Table 2:** Examination-based Demographics for Long-Term Risk Setting—Secondary Analysis

Demographic	Cancer Prior	Healthy
Age (y)		
40–55	477/1085 (44.0)	2267/4058 (55.9)
>55	608/1085 (56.0)	1791/4058 (44.1)
Density		
BI-RADS A	25/1085 (2.3)	109/4058 (2.7)
BI-RADS B	367/1085 (33.8)	1618/4058 (39.9)
BI-RADS C	571/1085 (52.6)	2021/4058 (49.8)
BI-RADS D	122/1085 (11.2)	310/4058 (7.6)
Biopsy		
Invasive	954/1085 (87.9)	0
In situ	107/1085 (9.9)	0
Unknown	24/1085 (2.2)	0
Total		
Examinations	1085	4058
Women	590	4051

Note.—Data are proportion of examinations with percentage in parentheses. Breast Imaging Reporting and Data System (BI-RADS) density was assessed using Transpara software. Patients who underwent cancer prior examinations developed cancer after at least one screening round and up to 7 years after the initial examination. There was no individual overlap between the case and control groups.

### Overall Performance

The receiver operating characteristic curves for the detection and risk models in all settings are shown in Figure 2, and the AUCs are summarized in Table 3. In the detection setting, in which positive cases included both screen-detected and interval cancers, there was no evidence of a difference in the performance of Transpara Detection (AUC, 0.92; 95% CI: 0.91, 0.93) and Transpara Risk (AUC, 0.92; 95% CI: 0.91, 0.93; adjusted *P* = .93). However, both models outperformed RSNA Detection (AUC, 0.90; 95% CI: 0.88, 0.91) and Mirai Risk (AUC, 0.88; 95% CI: 0.86, 0.89; all adjusted *P* < .001 compared with Transpara models). With respect to the subgroup comparison between in situ and invasive cancers, there was no evidence of a difference for Transpara Detection and Transpara Risk (*P* = .13 and .46, respectively). However, Mirai Risk and RSNA Detection performed better for in situ cancers than for invasive cancers (*P* < .001 and *P* = .004, respectively).

**Figure 2:**
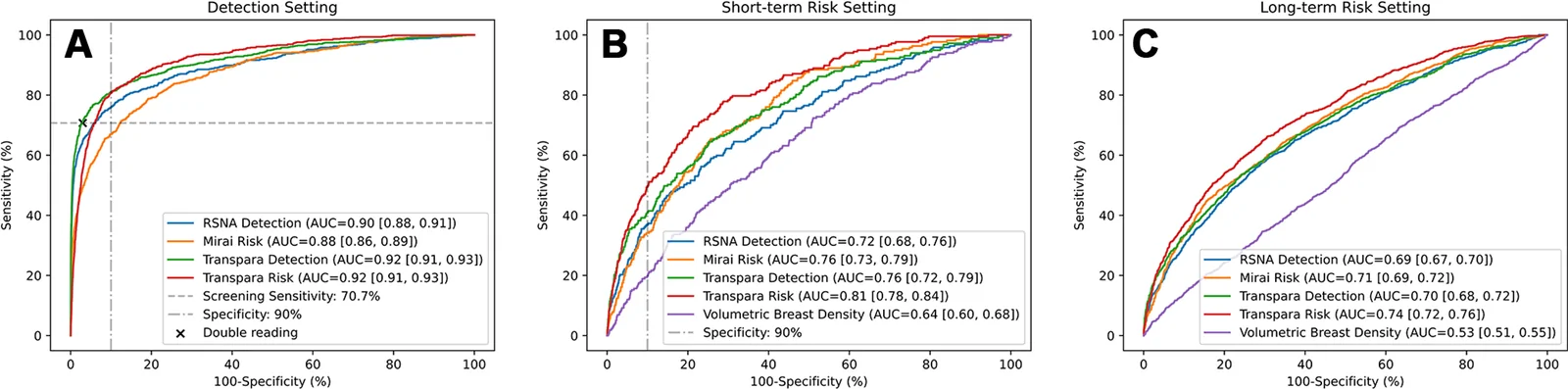
Graphs show the receiver operating characteristic curves for **(A)** detection, **(B)** short-term risk, and **(C)** long-term risk. Detection and risk models were also evaluated in their nonintended settings because of the overlapping nature of the detection and risk settings. Overall performance was evaluated using the area under the receiver operating characteristic curve (AUC) and is reported with 95% CIs. In the detection setting, double-reading performance is used as a reference; in both risk settings, volumetric breast density serves as the reference. Operating points were selected on the basis of double-read sensitivity and 90% specificity in the detection setting and 90% specificity in the detection and short-term risk settings.

**Table 3:** Discriminative Performance Measured with the AUC

Variable	No. of Positive/Negative Examinations	AUC (95% CI)			
		RSNA Detection	Mirai Risk	Transpara Detection	Transpara Risk
Detection setting					
All women	741/19 446	0.90 (0.88, 0.91)	0.88 (0.86, 0.89)	0.92 (0.91, 0.93)	0.92 (0.91, 0.93)
Biopsy					
Invasive	650/19 446	0.90 (0.88, 0.91)	0.87 (0.85, 0.88)	0.92 (0.91, 0.93)	0.92 (0.91, 0.93)
In situ	78/19 446	0.94 (0.91, 0.98)	0.95 (0.92, 0.98)	0.95 (0.92, 0.98)	0.93 (0.90, 0.97)
*P* value*	–	.004	<.001	.13	.46
Short-term risk setting					
All women	217/19 446	0.72 (0.68, 0.76)	0.76 (0.73, 0.79)	0.76 (0.72, 0.79)	0.81 (0.78, 0.84)
Biopsy					
Invasive	191/19 446	0.72 (0.68, 0.75)	0.75 (0.72, 0.78)	0.76 (0.72, 0.79)	0.82 (0.79, 0.84)
In situ	16/19 446	0.77 (0.65, 0.90)	0.83 (0.71, 0.95)	0.77 (0.65, 0.90)	0.78 (0.65, 0.90)
*P* value*	–	.38	.20	.85	.56
Long-term risk setting					
All women	1085/4058	0.69 (0.67, 0.70)	0.71 (0.69, 0.72)	0.70 (0.68, 0.72)	0.74 (0.72, 0.76)
Biopsy					
Invasive	954/4058	0.68 (0.67, 0.70)	0.70 (0.68, 0.72)	0.69 (0.68, 0.71)	0.74 (0.72, 0.75)
In situ	107/4058	0.67 (0.60, 0.73)	0.74 (0.69, 0.80)	0.72 (0.66, 0.77)	0.75 (0.70, 0.80)
*P* value*	–	.59	.12	.53	.69

Note.—AUC = area under the receiver operating characteristic curve.

* *P* value for the same model across different biopsy subgroups.

In the short-term risk setting, with positive cases including only interval cancers, Transpara Risk demonstrated the best performance, with an AUC of 0.81 (95% CI: 0.78, 0.84; all adjusted *P* < .001). There was no evidence of a difference between Transpara Detection and Mirai Risk, with AUCs of 0.76 (95% CI: 0.72, 0.79) and 0.76 (95% CI: 0.73, 0.79), respectively (adjusted *P* = .96). RSNA Detection had the lowest AUC of 0.72 (95% CI: 0.68, 0.76; all adjusted *P* = .01). As a reference, the volumetric breast density provided by Transpara achieved an AUC of only 0.64 (95% CI: 0.60, 0.68). No evidence of a difference between invasive and in situ cancers was observed for any model (all *P* ≥ .20), although this may be due to the small number of in situ cancer examinations (*n* = 16).

In the long-term risk setting, which focused on cancer priors up to 7 years, Transpara Risk achieved the best AUC of 0.74 (95% CI: 0.72, 0.76) (all adjusted *P* < .001). Notably, there was no evidence of a difference between Transpara Detection and Mirai Risk, with AUCs of 0.70 (95% CI: 0.68, 0.72) and 0.71 (95% CI: 0.69, 0.72), respectively (adjusted *P* = .44). Compared with Mirai Risk, RSNA Detection had a slightly lower AUC of 0.69 (95% CI: 0.67, 0.70; adjusted *P* = .01). There was no evidence of a difference in performance among the models across biopsy subgroups (all *P* ≥ .12).

### Selection of Operating Points in the Detection Setting

Model characteristics in both settings, after the operating points were set, are shown in Table 4. The operating points for the four models in the detection setting were first set to match the double-reading sensitivity of 70.7%. Transpara Detection achieved the highest specificity of 97.1% (95% CI: 96.8, 97.3). In contrast, compared with Transpara Detection, Transpara Risk, RSNA Detection, and Mirai Risk had lower specificities of 94.0% (95% CI: 93.6, 94.3), 94.2% (95% CI: 93.8, 94.5), and 87.8% (95% CI: 87.3, 88.2), respectively (all adjusted *P* < .001). No evidence of a difference was observed between Transpara Risk and RSNA Detection (adjusted *P* = .29).

**Table 4:** Characteristics after Setting Operating Points

Characteristic	RSNA Detection	Mirai Risk	Transpara Detection	Transpara Risk
Detection setting				
Double-reading sensitivity				
Specificity (%)	94.2 (93.8, 94.5)	87.8 (87.3, 88.2)	97.1 (96.8, 97.3)	94.0 (93.6, 94.3)
TN/N	18 309/19 446	17 072/19 446	18 879/19 446	18 270/19 446
TP (SDC/IC)	524 (468/56)	524 (439/85)	524 (470/54)	524 (440/84)
SDC/IC	8.4 (6.5, 11.5)	5.2 (4.1, 6.5)	8.7 (6.6, 11.8)	5.2 (4.2, 6.7)
90% specificity				
Sensitivity (%)	76.0 (72.8, 78.9)	66.9 (63.5, 70.2)	81.1 (78.1, 83.8)	80.7 (77.7, 83.4)
TP/P	563/741	496/741	601/741	598/741
TP (SDC/IC)	563 (483/80)	496 (422/74)	601 (512/89)	598 (490/108)
Short-term risk setting				
90% specificity				
Sensitivity (%)	36.9 (30.7, 43.5)	34.1 (28.1, 40.6)	41.0 (34.7, 47.7)	49.8 (43.2, 56.4)
TP/P	80/217	74/217	89/217	108/217

Note.—Data are AUCs with 95% CIs in parentheses or numbers out of the total numbers or numbers with subcategory numbers in parentheses. IC = number of examinations with interval cancers, N = number of pathology-confirmed negative examinations, P = number of pathology-confirmed positive examinations, SDC = number of examinations with screen-detected cancers, TN = number of true-negative examinations, TP = number of true-positive examinations.

To align with a recall rate of 10% in U.S. practice, operating points were also selected at a specificity of 90%. At this specificity, Transpara Detection and Transpara Risk achieved sensitivities of 81.1% (95% CI: 78.1, 83.8) and 80.7% (95% CI: 77.7, 83.4), respectively, with no evidence of a difference between them (adjusted *P* = .79). The sensitivities of RSNA Detection and Mirai Risk were lower, at 76.0% (95% CI: 72.8, 78.9) and 66.9% (95% CI: 63.5, 70.2), respectively (all adjusted *P* < .001). Transpara Risk outperformed RSNA Detection in terms of flagged screen-detected cancers (490 vs 483 cancers), even though it was designed for risk prediction.

### Tendencies for Identified Cancer Types at a Fixed Sensitivity

At the fixed sensitivity of 70.7% in the detection setting, the number of flagged cancerous examinations (524 examinations) was the same across all the models. However, because the positive cases included both screen-detected and interval cancers within the detection setting, the composition of the flagged cancer types varied across models. This variation reflects the different intended purposes of the models. Both detection models (Transpara Detection and RSNA Detection) had similar ratios of flagged screen-detected to interval cancers, at 8.7 (95% CI: 6.6, 11.8) and 8.4 (95% CI: 6.5, 11.5), respectively. In contrast, the risk models (Transpara Risk and Mirai Risk) had lower ratios of 5.2 (95% CI: 4.2, 6.7) and 5.2 (95% CI: 4.1, 6.5), respectively, indicating that they focused more on interval cancers than the detection models.

### Selection of Operating Points in the Short-Term Risk Setting

In the short-term risk setting at 90% specificity, Transpara Risk achieved the best sensitivity of 49.8% (95% CI: 43.2, 56.4) for interval cancers, outperforming Transpara Detection (sensitivity, 41.0%; 95% CI: 34.7, 47.7), RSNA Detection (sensitivity, 36.9%; 95% CI: 30.7, 43.5), and Mirai Risk (sensitivity, 34.1%; 95% CI: 28.1, 40.6; all adjusted *P* ≤ .002). The sensitivities of RSNA Detection and Transpara Detection for interval cancers were similar to that of Mirai Risk (both adjusted *P* ≥ .10), despite being primarily detection models and operating out of their intended use. As a reference, the Transpara volumetric breast density model achieved a sensitivity of only 19.8% (95% CI: 15.1, 25.6), which was lower than that of the other models, with no overlap in the 95% CIs.

### Agreement between the Detection and Risk Models

Transpara Detection and Transpara Risk were selected to investigate the agreement for screen-detected and interval cancers because of their superior performance in their respective settings. The scatterplot of all examinations based on prediction scores from both Transpara models (Fig 3), with both operating points set at a relatively high specificity of 90%, reveals that the majority of screen-detected cancers (93.1%; 488 of 524) were agreed on by both models. However, 4.7% (24 of 512) of screen-detected cancers identified by the detection model were not flagged by the risk model.

**Figure 3:**
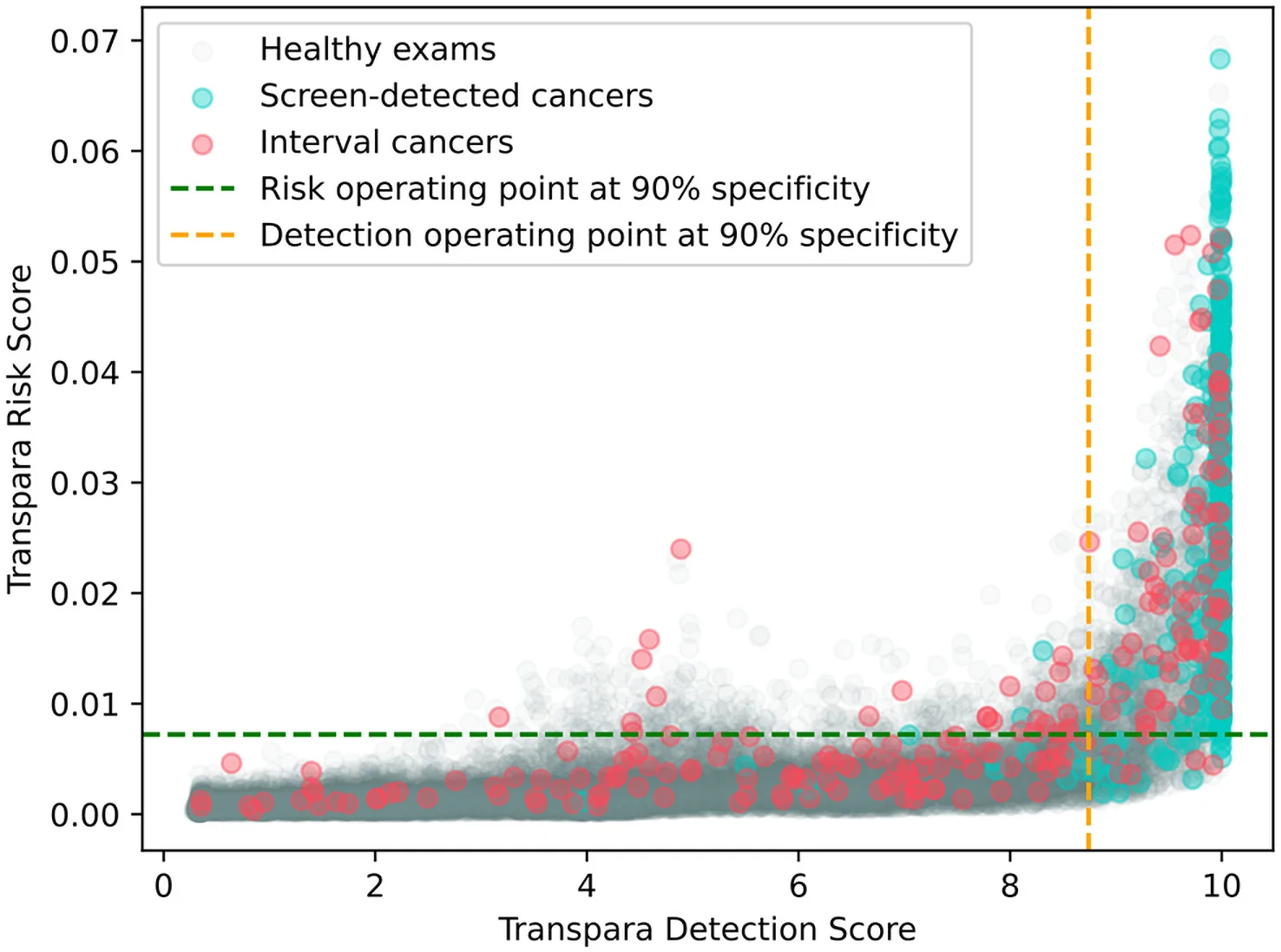
Scatterplot of all examinations based on Transpara Detection and Transpara Risk scores. Each examination is colored as healthy (gray), screen-detected cancer (blue), or interval cancer (red). The operating points of the two models at 90% specificity are shown as dashed lines.

With respect to interval cancers, the high agreement (38.7%; 84 of 217) between both models suggests that a subset of these examinations already exhibited lesion signs. Furthermore, following the clinical practice of applying a detection model before a risk model, only 11.1% (24 of 217) of interval cancers were identified by the risk models without being flagged by the detection model. Finally, 47.9% (104 of 217) of interval cancers were still missed by both models.

## Discussion

The performance of AI-based short-term mammographic risk models is heavily influenced by subtle lesion signs that may be missed or not considered actionable, raising questions about whether detection and risk models could be used interchangeably. In this study, detection and risk models were compared in both detection (all screening examinations) and short-term risk (screen-negative examinations only) settings to clarify their roles and overlap in a European screening round. In the detection setting, Transpara Detection and Transpara Risk outperformed the other models, with AUCs of 0.92 (95% CI: 0.91, 0.93; all adjusted *P* < .001) and no evidence of a difference between them (adjusted *P* = .93). However, the specificity of Transpara Detection was higher than that of Transpara Risk at the double-reading sensitivity. In the short-term risk setting, when only interval cancers were considered, Transpara Risk achieved the best AUC of 0.81 (95% CI: 0.78, 0.84), with the highest sensitivity of 49.8% (95% CI: 43.2, 56.4) at 90% specificity (all adjusted *P* ≤ .002). Interestingly, there was no evidence of a difference between Transpara Detection and Mirai Risk, with AUCs of 0.76 (95% CI: 0.72, 0.79) and 0.76 (95% CI: 0.73, 0.79), respectively (adjusted *P* = .96).

The model design and training approach may introduce biases. Lesion-biased risk prediction models, such as Mirai Risk, may overlook subtle texture patterns (18,19). However, including all screening examinations in the training process of Mirai Risk might help the model learn the lesion growth process, including patterns not visible to radiologists (28). The results of this study revealed that with the current model capabilities, risk models biased toward lesion signs may perform suboptimally in both short- and long-term risk settings. Notably, in the long-term risk setting, when diagnoses within one screening round were excluded, Transpara Detection still performed similarly to Mirai Risk. Additionally, both end-to-end training models (RSNA Detection and Mirai Risk) were biased toward detecting in situ cancers in the detection setting. This bias may be because information about cancer types was unavailable during training, causing the models to focus on in situ cancers, which often present as high-contrast, easily detectable calcifications.

Clinical research on the lesion visibility of prior screen-negative mammograms has demonstrated the inseparable relationship between lesion detection and risk prediction (4,15). This result underpins the use of computer-aided detection systems as short-term risk predictors (29). Conversely, Mirai Risk, which was trained using all screening examinations, could be applied in both settings (10). Although the line between detection and risk models has become blurred, their fundamental difference is their tendency to identify either screen-detected or interval cancers at the same sensitivity level, regardless of their discriminative power. This distinction is crucial because it informs how AI scores are defined and assigned in clinical practice. For a general definition of breast cancer risk (ie, the probability of cancer presence regardless of screening status), a screen-positive examination should be assigned a higher risk than examinations that are later followed by an interval cancer diagnosis because the former already indicates the presence of lesions. However, in practice, the radiologic characteristics of screen-detected and interval cancers differ, leading to different optimization purposes. Our results suggested that the best models for each setting were those optimized for their respective tasks.

The current consensus is to apply risk models to screen-negative examinations and to select women at high risk for supplemental screening (3). This approach guided the development of the leading risk model in this study, Transpara Risk, which is designed to operate exclusively in the risk setting. Because Transpara Risk and Transpara Detection had similar AUCs in the detection setting, we explored whether risk models could aid radiologists in reading borderline screening examinations. However, Transpara Risk provided low scores for some screen-detected cancers flagged by Transpara Detection, suggesting that this model might not be suitable as a recall decision support tool. In the off-label evaluation of detection models within short-risk settings, RSNA Detection and Transpara Detection performed comparably to Mirai Risk at clinically relevant specificity levels. This result raises the question of whether the 5% recall rate in European screening is too conservative. Examinations flagged by detection models contain known suspicious regions that could guide further evaluation with other modalities. Furthermore, the comparable performance of the detection and risk models in long-term settings might suggest that risk models, when influenced by lesion signs, can potentially identify cancers with longer sojourn times. In future research, such bias could be verified to ensure that risk models focus on identifying aggressive cancers.

This study has limitations. First, owing to the case-control design of the original dataset, cancer cases were curated across multiple screening rounds, and priors from the same woman were treated as independent healthy controls. However, the use of a publicly available dataset enables future comparisons. Second, the standalone evaluation of the detection and risk models may not reflect real clinical performance, as radiologist–AI collaboration has been shown to improve the standalone performance of such models (30). The study findings provide an initial benchmark for future prospective studies. Third, the performance of the Transpara models might be underestimated because the imputed age and compressed breast thickness, which were not available in the dataset, are important for risk prediction and density estimation, respectively. Finally, the generalizability of the results is limited by the use of a single mammography vendor and a single screening population. Specifically, the screening interval of 18 to 24 months differed from the standard biennial intervals used in other European countries. Because cancer characteristics are known to differ across screening intervals, further validation with other screening programs is warranted.

In conclusion, the benchmarking of AI-based breast cancer models in this study confirmed that detection models and risk models were better at identifying screen-detected and interval cancers, respectively. Despite the case-control design and standalone evaluation, this study provides a foundation for research on the integration of detection and risk models in screening contexts. Prospective studies and external validation are needed to assess patient outcomes in real-world settings.

## Supplemental Files

Appendix S1

Conflicts of Interest
