# Supplementary material for: Comparison of Artificial Intelligence Detection and Short-Term Breast Cancer Risk Models within and beyond Their Intended Use in Mammography Screening
Source: Radiol Imaging Cancer. 2026 Sep 25;8(5):e250609. doi: 10.1148/rycan.250609 (PMC13620329; doi:10.1148/rycan.250609)
Supplement: Supplementary file 1 [file rycan250609supp.pdf]

Supplemental materials undergo peer review but are not copyedited, and may include typographical errors.

©RSNA, 2026
10.1148/rycan.250609

Comparison of AI Detection and Short-Term Breast Cancer Risk Models Within and Beyond Their Intended Use in Mammography Screening

Supplemental Materials

Appendix S1: Data imputation for Transpara models

In addition to mammograms, Transpara models require breast thickness for volumetric breast density estimation and age at examination for risk calibration. However, the CSAW-CC dataset completely removes breast thickness from the DICOM files, while age is categorized as either 40–55 years or over 55 years. To obtain Transpara predictions, missing values for age and breast thickness were imputed. Age was imputed as the median value for each age group, 48 and 63 years, using rounded midpoints for the 15-year intervals.

Sensitivity test on breast thickness

To evaluate the effect of using a fixed breast thickness value on volumetric breast density, an analysis was conducted using in-house intermediate risk data comprising 463 women and 1,852 mammograms. The breast thickness distribution in this cohort demonstrated a mean of 60.6 mm and a median of 61.0 mm, with slight difference between craniocaudal (CC) and mediolateral oblique (MLO) views (mean thickness for CC: 59.8 mm; MLO: 61.5 mm).

For further assessment, a random selection of 30 examinations from the dataset was used. The breast thickness in these examinations was set to fixed values of 40 mm, 60 mm, and 80 mm, respectively, while all other DICOM tags were reduced to the minimal set required for processing by the Transpara model. The resultant breast density values from these fixed thickness groups were then compared to the original density measurements.

The differences between volumetric breast density with fixed thickness and the original density measurements were as follows: 0.87 ± 0.86 for 40 mm, 0.01 ± 0.87 for 60 mm, and -0.55 ± 0.77 for 80 mm. These results indicate that the breast density estimates are largely robust to inaccuracies in the breast thickness parameter, with only minimal impact observed when using a fixed value, particularly when the fixed thickness is near the mean of the original dataset. Finally, all breast thickness values were imputed as 61 mm (the median value of this in-house cohort).

Sensitivity test on prediction uncertainty

To investigate the influence of single-value imputations on the prediction uncertainty of Transpara Risk, we conducted random imputation 10 times for both age and breast

Supplemental materials undergo peer review but are not copyedited, and may include typographical errors.

thickness, aggregating the area under the receiver operating characteristic curve (AUC) using Rubin's rules.

Specifically, for each of the 10 iterations, breast thickness was randomly sampled from the above-mentioned in-house distribution for each examination, with four mammograms in the same examination sharing the same thickness value. Age was randomly sampled from either 40-54 or 55-69 years based on the age group of each examination.

Finally, the aggregated AUCs for the detection, short-term risk, and long-term risk settings were 0.92 (95% CI: 0.91, 0.93), 0.81 (95% CI: 0.78, 0.84), and 0.74 (95% CI: 0.72, 0.75), respectively, which were consistent with the AUCs obtained using single-value imputation (detection: 0.92 [95% CI: 0.91, 0.93]; short-term risk: 0.81 [95% CI: 0.78, 0.84]; long-term risk: 0.74 [95% CI: 0.72, 0.76]). We used the single-value imputation results in the main manuscript for consistency and simplicity.
